# Dissection of tumour and host cells from target organs of metastasis for testing gene expression directly ex vivo.

**DOI:** 10.1038/bjc.1996.519

**Published:** 1996-10

**Authors:** M. Rocha, K. Hexel, M. Bucur, V. Schirrmacher, V. Umansky

**Affiliations:** Tumor Immunology Program, German Cancer Research Center, Heidelberg, Germany.

## Abstract

**Images:**


British Journal of Cancer (1996) 74, 1216-1222
© 1996 Stockton Press All rights reserved 0007-0920/96 $12.00

### Dissection of tumour and host cells from target organs of metastasis for testing gene expression directly *ex vivo*

M Rocha, K Hexel, M Bucur, V Schirrmacher and V Umansky

*Tumor Immunology Program, Division of Cellular Immunology, German Cancer Research Center, Im Neuenheimer Feld 280, D-69120 Heidelberg, Germany.*

**Summary** We report on a new methodology which allows the direct analysis *ex vivo* of tumour cells and host cells (lymphocytes, macrophages, endothelial cells) from a metastasised organ (liver or spleen) at any time point during the metastatic process and without any further *in vitro* culture. First, we used a tumour cell line transduced with the bacterial gene *lacZ*, which permits the detection of the procaryotic enzyme $\beta$-galactosidase in eukaryotic cells at the single cell level thus allowing flow adhesion cell sorting (FACS) analysis of tumour cells from metastasised target organs. Second, we established a method for the separation and enrichment of tumour and host cells from target organs of metastasis with a high viability and reproducibility. As exemplified with the murine lymphoma ESb, this new methodology permits the study of molecules of importance for metastasis or anti-tumour immunity (adhesion, costimulatory and cytotoxic molecules, cytokines, etc.) at the RNA or protein level in tumour and host cells during the whole process of metastasis. This novel approach may open new possibilities of developing strategies for intervention in tumour progression, since it allows the determination of the optimal window in time for successful treatments. The possibility of direct analysis of tumour and host cell properties also provides a new method for the evaluation of the effects of immunisation with tumour vaccines or of gene therapy.

**Keywords:** lymphoma; tumour–host interaction; *ex vivo* analysis; genetic change

The interaction between tumour and host cells determines to a large extent the outcome, namely tumour growth and progression towards metastases or tumour arrest, dormancy or rejection. Most of the studies published so far on interactions of tumour cells and host cells were made *in vitro* and dealt with aspects such as cell adhesion, proliferation, invasiveness, cytotoxicity or cytokine production. Since the microenvironment in tissue culture differs in many respects from that *in vivo*, new approaches for *in vivo* studies of tumour–host cell interactions are of utmost importance in cancer research.

To elucidate the metastatic phenotype, approaches have been made to relate, for instance, cell surface molecules expressed on the tumour cell lines from tissue culture to their propensity to generate metastases *in vivo* (Nicolson 1982, 1987). Several authors have reported that certain steps of the metastatic cascade are rate limiting (Hart *et al.*, 1989; Fidler, 1990; Fidler and Radinski, 1990; Kerbel, 1990; Fodstad, 1993). To produce metastases, tumour cells must complete each of the sequential steps in the pathogenesis of cancer metastasis. Each discrete step appears to depend on the interaction between tumour cells and multiple host factors (i.e. the microenvironment of the tumour) and to be regulated by transient or permanent changes in DNA, RNA or proteins of multiple genes. Against this background, the need for comprehensive *in vivo*/*ex vivo* studies on tumour–host interactions and their kinetics in relevant model systems becomes obvious.

We have established a new method allowing the *ex vivo* isolation of tumour and host cells (tumour microenvironment) at any time point during the metastatic process and without any further *in vitro* culture. We chose as a model the ESbL lymphoma, transduced with the *lacZ* gene. This allows the detection of tumour cells at the single cell level either in frozen sections from target organs (liver, spleen, etc.) by staining with 5-bromo-4-cloro-3 indoyl-$\beta$-D-galactopyranoside (X-Gal) or as live cells after tissue dissociation staining with fluorescein-di-$\beta$-galactopyranoside (FDG) (Krüger *et al.*, 1994*a*). In this ESbL-*lacZ* tumour model, intradermal tumour growth and liver metastasis development followed three distinct phases: a first exponential growth phase, a transient plateau phase and a second expansion phase. The plateau phase was characterised by a constant tumour diameter, correlating with a constant low amount of metastasis in the liver. This phase was followed by an aggressive second expansion phase leading to macroscopic metastases in multiple visceral organs and to death of the animals within a few days (Krüger *et al.*, 1994*b*). These different phases of the metastatic process provide a good model for the investigation of the influence of tumour–host interactions during metastasis and of ways of modulating it. The plateau phase allows the study of the mechanisms of immunoresistance and tumour control. The second tumour expansion phase allows the study of the mechanisms of breakdown of immunoresistance and perhaps molecules of importance in metastatic progression.

### Materials and methods

### *Mice, cell lines and cell injections*

DBA/2 mice were obtained from Iffa Credo (Lyon, France) and used at 6–12 weeks of age. *lacZ*-transduced ESbL cells (clone L-CI.5s) were cultured as described (Krüger *et al.*, 1994*a*). Cells were washed in phosphate-buffered saline (PBS) and adjusted to the appropriate concentration. For standard intradermal injection, $2 \times 10^5$ cells were injected into the animal's cutis at the shaved flank of anaesthetised [Rompun (0.1%)–Ketanest (0.25%)–PBS diluted 1:1:3 (vol)] animals.

### *Isolation of tumour and sinusoidal cells from metastatic livers*

Cell isolation was performed as described (Rocha *et al.*, 1996). Briefly, livers from anaesthetised tumour-bearing mice were washed *in situ* by perfusion through the portal vein at 37°C with 10 ml $\alpha$ modified Eagle medium (MEM) containing 15 mM Hepes at a flow rate of 3 ml min$^{-1}$. Tissue digestion was carried out during perfusion with 10 ml of $\alpha$-MEM/Hepes containing 0.05% pronase E (Boehringer Mannheim, Germany) at 1 ml min$^{-1}$ and then with 15 ml

Correspondence: M Rocha
Received 6 February 1996; revised 19 April 1996; accepted 24 April 1996

of the same medium containing 0.03% pronase E, collagenase A (from *Clostridium hystolyticum*, Boehringer Mannheim). After perfusion, livers were minced and stirred in 13 ml α-MEM/Hepes containing 0.04% pronase E, 0.04% collagenase and 0.0004% DNAase (Sigma Chemical Co., USA) at 37°C for 10 min. The cell suspension was then filtered through a nylon gauze and centrifuged at 300 ***g*** for 10 min. To remove cell debris and erythrocytes, the cell pellet was centrifuged at 1400 ***g*** for 15 min in α-MEM/Hepes containing 17.5% (w/v) metrizamide (Sigma Chemical Co.), followed by washing of the top layer with α-MEM/Hepes at 300 ***g*** for 10 min.

*Antibody staining of tumour and sinusoidal cells*

About $1 \times 10^6$ cells were washed in PBS buffer supplemented with 5% fetal calf serum (FCS) and incubated at 4°C for 10 min with first antibodies. The following rat anti-mouse monoclonal antibodies were used as culture supernatants: anti-CD4 (clone GK 1.5); E-selectin (clone 21KC10), specific for endothelial cells; and anti-macrophage antibody (F4/80). After washing, cells were incubated with the second antibody ($F(ab')_2$ goat anti-rat, mouse Ig absorbed, R-phycoerythrin conjugated, Gibco BRL). Control cells were incubated with FACS buffer instead of the antibody, before staining with the second antibody.

*FDG staining and FACS analysis of tumour and host cells*

After antibody staining, sinusoidal cells were washed in PBS supplemented with 5% FCS and incubated in 100 ml PBS/FCS at 37°C for 10 min. Quantification of liver metastases was performed at the single cell level by loading and staining with FDG (fluorescein-di-β-D-galactopyranoside) as described (Krüger *et al.*, 1994*b*). Cells were loaded by hypotonic shock with 100 ml of prewarmed FDG (Molecular Probes, Inc., Eugene, OR, USA) in water, mixed, vortexed and incubated for 1 min at 37°C. Ice-cold 5% FCS/PBS (1.8 ml) was then added, the cells were kept for 10 min on ice and treated for 5 min before analysis with 1.5 μM propidium iodide (final concentration). Flow adhesion cell sorting (FACS) analysis was performed using a FACScan (Becton-Dickinson). About 30 000 cells per sample were simultaneously measured for FSC and integrated side scatter (SSC) as well as green (FL1) and red (FL2 and FL3) fluorescences (expressed as logarithm of the integrated fluorescence light). Recordings were made only on propidium iodide-negative (viable) cells of the red (FL3) fluorescence, excluding aggregates whose FSCs were out of range. *Ex vivo* expression of cell surface molecules was analysed by histograms of red fluorescence (FL2) distribution plotted as number of cells (*y*-axis) *vs* fluorescence intensity (*x*-axis) for the different tumour and sinusoidal cell populations.

*Isolation of liver endothelial cells*

After isolation of liver sinusoidal and tumour cells, they were cultured on type 1 collagen-coated plastic Petri dishes in α-MEM supplemented with 10% FCS at 37°C in an incubator under 5% carbon dioxide/air. Two hours later supernatants were collected and adherent cells (endothelial cells) were scraped off with a rubber spatula, counted, pelleted, snap frozen in liquid nitrogen and kept at −70°C for RNA studies. The same procedure was used for isolation of endothelial cells from normal (non-tumour-bearing) mice. Purity of endothelial cell populations was evaluated as described (Rocha *et al.*, 1995).

*Isolation of Kupffer cells from metastatic livers*

Kupffer cells were isolated after differential adhesion from the collected supernatants. Plastic culture dishes (Greiner, Germany) were treated with 2.5% glutaraldehyde (Merck, Germany) in PBS for 2 h at 4°C followed by washing 10 times in PBS and incubation of isolated sinusoidal cells for 30 min at 37°C in 5% carbon dioxide. The adherent cells were removed with a rubber spatula, counted, pelleted, snap frozen in liquid nitrogen and kept at −70°C for RNA studies. Purity of Kupffer cell population was evaluated as described (Umansky *et al.*, 1995).

*Isolation of lymphocytes and tumour cells from metastatic livers*

Separation of tumour cells from lymphoid cells was performed by FDG staining and flow cytometry cell sorting. FDG staining was performed as described (Krüger *et al.*, 1994*b*). About $5 \times 10^6$ cells per sample of isolated tumour and lymphoid cells which remained after the two adhesion steps described above, were washed in PBS with FCS and incubated at 37°C for 10 min. Then, 100 ml of prewarmed FDG (Molecular Probes, Eugene, OR, USA) in water were added to the cell suspension and incubated for 4 min at 37°C (hypotonic shock). Cold 5% FCS/PBS (1.8 ml) was then added, the cells kept for 10 min on ice and stained with 1.5 μM propidium iodide (final concentration). Sorting was performed using a FACS Vantage sorter (Becton-Dickinson, Heidelberg, Germany). A window for sorting was defined in the FDG-positive cells (tumour cells) excluding dead cells and debris with propidium iodide. Flow rate was 3000–5000 cells $s^{-1}$ and sorted cells were collected in sterile tubes containing RPMI with 20% FCS. After centrifugation of the total collected fractions, tumour cells and lymphocytes, pellets were snap frozen in liquid nitrogen and kept at −70°C until use for RNA extraction.

*RNA extraction, hybridisation and densitometric quantitation*

Cell pellets were homogenised with 0.2 ml of RNA-Clean (Angewandte Gentechnologie Systeme, Heidelberg, Germany) per $2 \times 10^6$ cells and RNA extraction performed by the chloroform/phenol technique. Extracted RNA was precipitated with isopropanol, the pellet washed in ethanol, dried under vacuum and resuspended in DEPC treated water. Quantity of RNA was measured by absorbance at 260 nm. Total isolated RNA was denatured and spotted onto a nitrocellulose filter using a dot blot apparatus and fixed by UV cross-linking with vacuum (Heraeus). Membranes were prehybridised for 2 h at 42°C in solution containing 1% formamide, 20 × saline sodium citrate (SSC) and 1% sodium dodecyl sulphate (SDS). Hybridisation was performed in the same solution for 24–48 h at 42°C with the cDNA probes. The following cDNA probes were used for hybridisation: MHC class II, α and β chain (kindly provided by Dr F Momburg), ICAM-1 and integrin α4 chain (kindly provided by Dr P Altevogt). cDNA inserts were labelled with $^{32}P$ to a specific activity of about $2 \times 10^8$ c.p.m. per μg DNA by oligolabelling kit (Pharmacia, Sweden). After hybridisation, filters were washed three times for 30 min with SSC and SDS at 68°C. Membranes were exposed to O-MAT films (Kodak, Germany) at −70°C. Expression of the mRNA was quantified by densitometry of autoradiograms using the Adobe Photoscop Program and the SCAN analysis program from Macintosh with each sample measurement calculated from the ratio of the average areas between the specific mRNA transcripts and the β-actin mRNA transcripts.

### Results

*Identification and analysis of* ex vivo *isolated tumour and sinusoidal cells during lymphoma metastasis by flow cytometry*

The number of sinusoidal cells obtained per mouse liver was $5-8 \times 10^6$ depending on factors such as age and weight of the mice and size of the liver. In the case of metastatic livers, the total amount of sinusoidal and tumour cells was $20-25 \times 10^6$ per mouse. Viability was higher than 93% in both cases. After antibody incubation and FDG staining, we analysed tumour cells and sinusoidal cells by flow cytometry. Figure 1 shows the analysis of sinusoidal and tumour cells isolated from metastatic livers at day 28 after tumour injection. At this time point the percentage of tumour cells in the liver is 40–50% of the total sinusoidal cell population (Krüger *et al.*, 1994*b*). Contour plots of the total population were analysed following loading and staining with FDG. Tumour cells were identified as green fluorescence-positive cells (R2) and compared with the non-fluorescent cells, which represent the sinusoidal cell population (R1). Different sinusoidal cell populations were identified as previously described (Rocha *et al.*, 1995) and gated for separate analysis. Further characterisation of the gated populations was achieved by cell surface staining with rat monoclonal antibodies (MAbs) and dye-labelled secondary anti-rat Ig reagents. As can be seen from the profiles in Figure 1, CD4 was positive in the first region (lymphoid cells), E-selectin in the second region (endothelial cells) and F4/80 in the third region (Kupffer cells). Tumour cells could be easily distinguished as FDG-positive cells (Figure 1), although the hypotonic shock treatment also slightly changed the profiles of the normal cell populations. Combining FDG staining of tumour cells and MAb staining, we recently demonstrated up-regulation of the expression of surface molecules, such as LFA-1 and ICAM-1, on liver metastases. Further antibody blocking experiments suggested that these adhesion molecules were of importance for metastatic expansion in the liver (Rocha *et al.*, 1996).

### *Isolation and enrichment of tumour and host cell populations from metastatic organs for studies at the transcriptional level*

After *in vivo* liver perfusion and metrizamide gradient centrifugation, the isolated total sinusoidal cell population

**Figure 1** Flow cytometric analysis of *ex vivo* isolated cells from a metastasised liver, 28 days after intradermal transplantation of ESb1-*lac*Z lymphoma cells. **a** and **b**, Contour plots of forward scatter *vs* green fluorescence (FL1) of isolated cells without (a) or with (b) FDG loading. Tumour cells were identified in **b** as green fluorescence-positive cells (R2) and compared with normal host sinusoidal cells (R1). R3 represents the total population. **c**, Two-parameter slice plots of forward side scatter of the sinusoidal population from **b**. R4 represents lymphocytes, R5, endothelial cells and R6, Kupffer cells. Identity of the gated populations, R4–R6, is shown by the FACS profiles below after indirect immunofluorescence staining with CD4 (positive in R4, lymphoid cells), E-selectin (positive in R5, endothelial cells) and F4/80 (positive in R6, Kupffer cells). Tumour cells were identified as fluorescence positive after hypotonic loading with FDG. Controls included cells with or without FDG loading or with and without first antibody. In each column, the left histogram represents the non-specific binding of PE-conjugated second antibody and the right histogram the specific binding of the indicated MAb to the same cells. When two histograms match, specific binding is negligible or absent. Numbers indicate the percentage of positive cells with the respective antibodies.

was subfractionated into different populations. The percentage recovery of cells at the end of the whole procedure was 50–60%. First, cells were plated on collagen-pretreated dishes to separate adherent endothelial cells. Two hours was found to be the optimum culture time in terms of viability, purity and yield of endothelial cells. The number of cells obtained was approximately 25–35% of the total seeded population (Figure 2). The non-adherent cells were removed and seeded over glutaraldehyde-pretreated dishes, which has been described as being an effective method to obtain Kupffer cells with a high purity (Smedsrod *et al.*, 1985). Since the viability of the separated population decreased dramatically with time in culture, we decided that 30 min was the optimal time in terms of cell yield and viability. The number of Kupffer cells obtained was between 35% and 40% of the total seeded population (Figure 2). Both endothelial and Kupffer cell fractions were scraped off with a rubber policeman, pelleted and snap frozen to avoid RNA destruction by RNAases (Figure 2).

The remaining non-adherent cell suspension was a mix of lymphocytes and *lac*Z-tagged tumour cells. FDG loading of the cell suspension and FACS cell separation was performed as described in Materials and methods. At day 28 after intradermal ESbL-*lacZ* tumour cell inoculation, the number of tumour cells in the liver approximated 15–25% of the total population and that of the lymphocytes about 10–15% (Figure 2).

As a second metastasised organ we investigated the spleen. Spleen macrophages were isolated from the single cell suspension by plastic adherence. The remaining supernatant, containing lymphocytes and tumour cells, was stained with FDG and sorted as described above.

Figure 3 shows examples of the analysis of gene expression of different cellular subpopulations isolated from metastatic spleens or livers. Lymphocytes isolated from control spleens and from metastatic spleens at the plateau phase (day 16) and at the end of the metastatic process (day 28) showed differences at the RNA level with regard to expression of distinct molecules, such as MHC class II α chain (Figure 3a and b). The signal obtained with MHC class II α chain cDNA or other probes was compared with β-actin as internal standard and transformed into expression units. Lymphocytes at day 28 of the metastatic process (column 3) expressed only 46% of the α chain in comparison with lymphocytes from normal control mice (column 1). Spleen macrophages isolated at day 28 (row 4) expressed 80% class II α chain in comparison with controls (column 2). Column 5 shows the expression of MHC class II α chain in splenic lymphocytes and macrophages together from day 28 metastasised organs and column 6 represents the expression of class II α chain by the sorted tumour cells.

Liver endothelial cells were isolated from metastatic livers at day 16 or day 28 after tumour cell inoculation. The expression of different RNA molecules was compared with cells from two different experiments and time points (day 16, columns 8 and 9; day 28, column 10). As shown in Figure 3c and d, expression of ICAM-1 was down-regulated at day 16 in both experiments (40% and 35% respectively) and less so at day 28, compared with the control. This differential expression if ICAM-1 in endothelial cells isolated from metastatic livers at different time points has been observed previously also at the protein level (Rocha *et al.*, 1996).

Analysis of Kupffer cells (Figure 3e and f), isolated at the same time points (day 16 and 28) showed no significant changes in the expression of ICAM-1 molecules. The study of the expression of MHC class II molecules (β-chain) in Kupffer cells from day 16 (columns 12 and 13) and day 29 (columns 14 and 15) compared with the control (column 11) showed that there was a slight up-regulation of expression of this molecule at day 16 after tumour injection.

Isolated tumour cells were studied for the expression of different molecules potentially involved in metastasis formation. We found differences, as shown in Figure 3g and h, in expression of VLA-4 RNA in tumour cells from tissue culture (column 17) and that of tumour cells from day 16 (column 18) or day 28 (column 19). Results show that the expression of VLA-4 is down-regulated in tumour cells *in vivo* at both time points. To prove that the FDG technique did not miss some *in vivo* revertant tumour cells, we performed a control experiment as follows: we isolated host and tumour cells from metastatic livers at day 28, when tumour load is 50% of the total population. After long-term culture only tumour cells should survive. Therefore, if the *in vivo* isolated cells kept the *lac*Z gene, all the remaining tumour cells in the culture should have it, since it is genetically transferred. If after this time in culture we found FDG-negative cells, it would mean that some of these tumour cells lost the *lac*Z gene during *in vivo* development.

**Figure 2** Method of *ex vivo* separation of cells from a metastasised liver for preparation of total RNA. Sinusoidal cells and tumour cells from metastatic livers were fractionated into subpopulations by differential adhesion (endothelial and Kupffer cells) and by FACS (lymphocytes and tumour cells). Numbers indicate the range in percentage of cells obtained in each purification step relative to the total number of live recovered cells.

a

1

2

3

4

5

6

B

Spleen cells

MHC class II (α)

Units

LYM CONTROL

MAC CONTROL

LYM DAY 28

MAC DAY 28

HOST CELLS

TUMOR CELLS

c

7

8

9

10

D

Liver endothelial cells

ICAM-1

CONTROL

DAY 16

DAY 16

DAY 28

e

11

12

13

14

15

F

Kupffer cells

MHC class II (β)

CONTROL

KC DAY 16

KC DAY 16

KC DAY 28

KC DAY 28

g

17

18

19

Probe

β-actin

H

Tumour cells

VLA-4

CONTROL

TC DAY 16

TC DAY 28

FDG staining after one culture showed that 99.5% of the cells were FDG-positive cells and no revertant cells were missed by the FDG technique.

In conclusion, our method provides a new tool to study expression changes at the RNA and protein level of molecules potentially involved in metastasis progression or inhibition.

### Discussion

Organotropism, i.e. the preference of certain cancers to metastasise to certain organs and tissue-specific metastasis patterns are quite often seen in clinic samples and in experimental tumour models. Both phenomena underline the importance of local microenvironmental factors for the development of metastases (Fidler, 1986; Liotta, 1986; Hoffman, 1992). Several studies correlated phenotypic features of tumour cells before being injected into animals with the final outcome of metastasis and formed the basis for terms such as the 'metastatic phenotype' (Kerbel 1990; Fidler, 1990). Also, in recent years the concept of 'dynamic heterogeneity' was introduced. It suggests that the metastatic phenotype, although it is a genetically controlled trait, is inherently dynamic or unstable (Weiss *et al.*, 1980; Ling *et al.*, 1985; Vaage, 1988). Because of this phenotypic instability, an adequate methodology has to be established for direct typing of tumour cells at distinct stages of the metastatic process without *in vitro* culture.

We have established a new methodology for the study of tumour–host interactions in the metastatic process. Firstly, we used a tumour cell line transduced with the bacterial gene *lac*Z, to study tumour–host interactions, for instance during micrometastasis, minimal residual disease or tumour dormancy. Secondly, we have established a method allowing the separation of tumour and host cells from the liver and spleen with a high viability (93–95%) and reproducibility which can be used for different target organs. This permits the direct characterisation of *ex vivo* isolated cells without further *in vitro* culture. Control experiments in reisolated and cultured tumour cells showed that the FDG technique is very specific and sensitive, since all the tumour cells express the *lac*Z gene after being grown *in vivo*. This excludes the possibility of the existence of *lac*Z-revertant tumour cells which could not be detected and quantified as tumour cells.

The described technique provides the possibility of studying molecules involved in the metastatic process at two different levels, mRNA and protein (for instance, adhesion molecules, homing receptors, immune stimulatory molecules, etc.). Malignant cells disseminate from a locally growing tumour to other sites by means of adhesion molecules (e.g. LFA-1, ICAM-1, VLA-4) and homing receptor molecules (e.g. MEL-14) which are used by haemopoietic normal cells for traffic and localisation in various organs or at sites of inflammation. It has been suggested, for example, that the adhesion of melanoma cells to activated endothelium is mediated by VLA-4 receptors, thus implicating this adhesion molecule in metastatic process (Garofalo *et al.*, 1995). Our results, however, showed that the VLA-4 molecule was down-regulated in the ESbL-*lac*Z tumour cells when injected into syngeneic animals. Evidence that LFA-1 and ICAM-1 are involved in metastatis has also been presented (Zalhaka *et al.*, 1993; Harning *et al.*, 1993). In support of these observations, we have recently shown by using this new methodology that the expression of LFA-1 and ICAM-1 molecules was up-regulated during the progressive phase of tumour growth and metastasis in the ESbL-*lac*Z lymphoma model (Rocha *et al.*, 1996). Consequently, a strategy was developed to apply antibodies against these adhesion molecules before the final progressive phase of tumour growth started. Such experiments resulted in complete inhibition of tumour progression *in vivo* (Rocha *et al.*, 1996).

The results from Figure 3 show that the expression of adhesion molecules such as ICAM-1 in endothelial cells or VLA-4 in metastasising tumour cells is regulated *in vivo* at the RNA level. This method of analysis may thus provide a possibility of determining which molecules and which window in time may be suited for therapeutical intervention.

Other molecules such as costimulatory molecules, MHC class I or class II and CD80 (B7-1), which have been reported to be important in the recognition of the tumour cells by the immune system (Becker *et al.*, 1993; Chen *et al.*, 1992), can also be studied by this method during the whole process of tumour growth and metastasis. The observed down-regulation of MHC class II RNA in lymphocytes from metastatic spleens at a late stage of metastasis could lead to a decrease of recognition of tumour-associated antigens on tumour cells, hampering an effective immune response.

Northern and slot blot techniques allowed the study of molecules of importance in metastasis. There have been numerous reports showing that the metastatic potential of tumours may correlate directly with the expression level of distinct genes. Some genes coded for adhesion and costimulatory molecules (Becker *et al.*, 1993; Chen *et al.*, 1992), others for growth factors, growth factor receptors, enzymes or multidrug resistance (bFGF, EGFR, type IV collagenase or mdr-1) (Fidler, 1995). In this study, the differential expression of genes coding for VLA-4, ICAM-1 and MHC class II on tumour and host cells was closely associated with metastatic progression or growth retardation.

The technique provides new possibilities of using *ex vivo* isolated cells for different purposes. *Ex vivo* isolated metastasised tumour cells can be used for cell–cell interaction studies with different host cells, for investigation of cytokine and growth factor production and as target cells in cytotoxicity assays with *ex vivo* isolated lymphocytes or macrophages. Endothelial and Kupffer cells provide a tool for adhesion studies with tumour cells (Asumendi *et al.*, 1996) and for evaluation of the production of different cytokines and cytotoxic molecules. One example of cytotoxic substances produced by activated host cells is nitric oxide (NO) (Rocha *et al.*, 1995; Umansky *et al.*, 1995). NO has been identified recently as an effector molecule of cytotoxicity mediated by macrophages and endothelial cells (Hibbs *et al.*, 1988). Its toxic effect is a result of inhibition of DNA synthesis and of

**Figure 3** Slot blot analysis of gene expression of different cellular subpopulations isolated from metastatic spleens or livers of Esb-*lac*Z tumour bearing mice. Organs were removed from either normal mice (control) or from tumour-bearing mice 16 days or 28 days after intradermal tumour cell transplantation. Tumour and host cells were separated and total RNA prepared as described in Figure 2. The different RNAs were blotted onto nitrocellulose and hybridised with test probes for MHC class II $\alpha$ chain (**a**), ICAM-1 (**c**), MHC class II $\beta$ chain (**e**), VLA-4 ($\alpha$4) (left panels) or with a $\beta$-actin probe as internal control (right panels in **a**, **c**, **e** and **g**. The slot blots are shown in **a**, **c**, **e** and **g** and the corresponding histograms of gene expression in **b**, **d**, **f** and **h**. Values in the histograms were obtained as follows: levels of mRNA expression were measured by densitometry and the ratio of densities between the expression of each test molecule and its corresponding $\beta$-actin control was determined. For every test probe the maximum value was considered as 100 units to which the other values were related. We evaluated mRNA transcripts in host and tumour cells separated from two independent experiments. Since results were comparable, we give the results of one experiment. *indicates significant differences in comparison with respective controls ($P<0.05$). In **b**, lym, lymphocytes; mac, macrophages; host cells, total spleen cells (day 28) and tumour cells, spleen-metastasised tumour cells (day 28). In **f**, KC, Kupffer cells. In **h**, gene expression in control tumour cells from tissue culture is compared with that of tumour cells (TC) from metastasised livers (day 16 and 28).

mitochondrial respiration in tumour target cells (Moncada *et al.*, 1991). Lymphocytes can be used to investigate cytokine production, in proliferation assays and as a tool for different therapeutical interventions.

In conclusion, we should like to point out the potential of this methodology. It allows the direct *ex vivo* analysis of gene expression (RNA or protein) in tumour and host cells at every time point during tumour growth and metastasis. This experimental approach opens a broad possibility for studies of the basic mechanisms of tumour development, for example: (1) evaluation of vaccination effects in target organs; (2) determination of the right window in time for cancer therapy; and (3) investigation of the effects of transferred immune cells, tissues or organs on the host, for instance in the processes of graft *vs* leukaemia and graft *vs* host disease (Schirrmacher *et al.*, 1995). Work is now in progress on the establishment of methods allowing the separation of host and tumour cells from human primary tumours and metastasis.

**Acknowledgements**

The authors wish to thank Dr P Altevogt (Division of Cellular Immunology, DKFZ) and Dr F Momburg (Division of Molecular Immunology, DKFZ) for providing us with the constructs for hybridisation. This work was partially supported by an EC grant (Human capital and mobility programme) (Dr Marian Rocha).

### References

ASUMENDI A, CALVO F, HERNANDEZ JJ, ALVAREZ A, ROCHA M AND VIDALVANACLOCHA F. (1996). Cancer cell surface mannose terminals and their receptors in liver and bone marrow sinusoidal endothelium contribute to B16 melanoma cell adhesion and metastasis. *Lab. Invest.* (in press).

BECKER JC, BRABLETZ T, CZERNY C, TERMEER C AND BROCKER EB. (1993). Tumor escape mechanisms for immunosurveillance: induction of unresponsiveness in specific MHC-restricted CD4+human T cell clone by the autologous MHC class II + melanoma. *Int. Immunol.*, **5**, 1501–1508.

CHEN L, ASHE S, BRADY WA, HELLSTROM I, HELLSTROM KE, LEDBETTER JA, MCGOVAN P AND LINSLEY PS. (1992). Costimulation of antitumor immunity by the B7 counterreceptor for the T lymphocyte molecules CD28 and CTLA-4. *Cell*, **71**, 1093–1098.

FIDLER IJ. (1986). Rationale and methods for the use of nude mice to study the biology and therapy of human cancer metastasis. *Cancer Metast. Rev.*, **5**, 29–49.

FIDLER IJ. (1990). Critical factors in the biology of human cancer metastasis: Twenty-eighth GHA Clowes Memorial Award Lecture. *Cancer Res.*, **50**, 6130–6138.

FIDLER IJ. (1995). Multiparametric *in situ* messenger RNA hybridization analysis to detect metastasis-related genes in surgical specimens of human colon carcinomas. *Clin. Cancer Res.*, **1**, 1095–1102.

FIDLER IJ AND RADINSKY R. (1990). Genetic control of cancer metastasis (editorial). *J. Natl. Cancer Inst.*, **82**, 166–168.

FODSTAD O. (1993). Metastatic ability of cancer cells: pheno- and genotypic characteristics and role of the micro-environment. In *New Frontiers in Cancer Causation*. Iversen OH (ed.) pp. 349–358. Taylor & Francis: Washington DC.

GAROFALO A, CHIVIRI RGS, FOGLIENI C, PIGOTT R, MORTARINI R, MARTIN-PADURA I, ANICHINI A, GEARING AJ, SANCHEZ-MADRID F, DEJANA E AND GIAVAZZI R. (1995). Involvement of the very late antigen 4 integrin on melanoma in interleukin 1-augmented experimental metastasis. *Cancer Res.*, **55**, 414–419.

HARNING R, MYERS C AND MERLUZZI VJ. (1993). Monoclonal antibodies to lymphocyte function-associated antigen-1 inhibit invasion of human lymphoma and metastasis of murine lymphoma. *Clin. Exp. Metast.*, **11**, 337–342.

HART IR, GOODE NT AND WILSON RE. (1989). Molecular aspects of the metastatic cascade. *Biochim. Biophys. Acta*, **989**, 65–84.

HIBBS JB, TAINTOR R, VAVRIN Z AND RYCHLIN EM. (1988). Nitric oxide: a cytotoxic activated macrophage effector molecule. *Biochem. Biophys. Res. Commun.*, **157**, 87–94.

HOFFMANN RM. (1992). Patient like models of human cancer in mice. *Current Persp. Mol. Cell. Oncol.*, **1**, 311–326.

KERBEL RS. (1990). Growth dominance of the metastatic cancer cell: cellular and molecular aspects. *Adv. Cancer Res.*, **55**, 87–132.

KRÜGER A, UMANSKY V, ROCHA M, HACKER HJ, SCHIRRMACHER V AND VON HOEGEN P. (1994*a*). Pattern and load of spontaneous liver metastasis dependent on host immune status studied with *lacZ* transduced lymphoma. *Blood*, **84**, 3166–3174.

KRÜGER A, SCHIRRMACHER V AND VON HOEGEN P. (1994*b*). Scattered micrometastais visualized at the single cell level: detection and re-isolation of *lacZ* labeled metastasized lymphoma cells. *Int. J. Cancer*, **58**, 1–10.

LING V, CHAMBERS AF, HARRIS JF AND HILL RP. (1985). Quantitative genetic analysis of tumor progression. *Cancer Metast. Rev.*, **4**, 173–194.

LIOTTA L. (1986). Tumor invasion and metastasis: role of extracellular matrix: Rhoads Memorial Award Lecture. *Cancer Res.*, **46**, 173–194.

MONCADA S, PALMER RJ AND HIGGS EA. (1991). Nitric oxide: physiology, pathophysiology and pharmacology. *Pharmacol. Rev.*, **43**, 109–134.

NICOLSON GL. (1982). Cancer metastasis: organ colonization and the cell surface properties of malignant cells. *Biochim. Biophys. Acta*, **695**, 113–176.

NICOLSON GL. (1987). Tumor cell instability, diversification and progression to the metastatic phenotype: From oncogene and oncofetal expression. *Cancer Res.*, **47**, 1473–1487.

ROCHA M, KRÜGER A, VAN ROOIJEN N, SCHIRMMACHER V AND UMANSKY V. (1995). Liver endothelial cells participate in T-cell dependent host resistance to lymphoma metastasis by production of nitric oxide *in vivo*. *Int. J. Cancer*, **55**, 405–408.

ROCHA M, KRÜGER A, UMANSKY V, VON HOEGEN P, NAOR D AND SCHIRRMACHER V. (1996). Dynamic expression changes of adhesion and costimulatory molecules determine pattern and load of lymphoma liver metastasis. *Clin. Cancer Res.* (in press).

SCHIRRMACHER V, BECKHOVE P, KRÜGER A, ROCHA M, UMANSKY U, FITCHNER KP, HULL W, ZANGEMEISTER-WITTKE U, GRIESBACH A, JURIANZ K AND VON HOEGEN P. (1995). Effective immune rejection of advanced metastasized cancer. *Int. J. Oncol.*, **6**, 505–521.

SMEDSROD B, PERTOFT H, EGGERTSEN G AND SUNDSTROM C. (1985). Functional and morphological characterization of cultures of Kupffer cells and liver endothelial cells prepared by means of density separation in Percoll, and selective substrate adherence. *Cell Tissue Res.*, **241**, 739–749.

UMANSKY V, ROCHA M, KRÜGER A, VON HOEGEN P AND SCHIRRMACHER V. (1995). *In situ* activated macrophages are involved in host resistance to lymphoma metastasis by production of nitric oxide. *Int. J. Oncol.*, **7**, 33–40.

VAAGE J. (1988). Metastasizing potentials of mouse mammary tumours and their metastases. *Int. J. Cancer*, **41**, 855–858.

WEISS L, ORR FW AND HORN KV. (1980). Interactions between cancer cells and the microvasculature: a rate-regulator for metastasis. *Clin. Exp. Metast.*, **7**, 127–167.

ZALHAKA MA, ÖKON E AND NAOR D. (1993). Blocking lymphoma invasiveness with a monoclonal antibody directed against the $\beta$ chain of leucocyte adhesion molecule (CD18). *J. Immunol.*, **150**, 4466–4477.


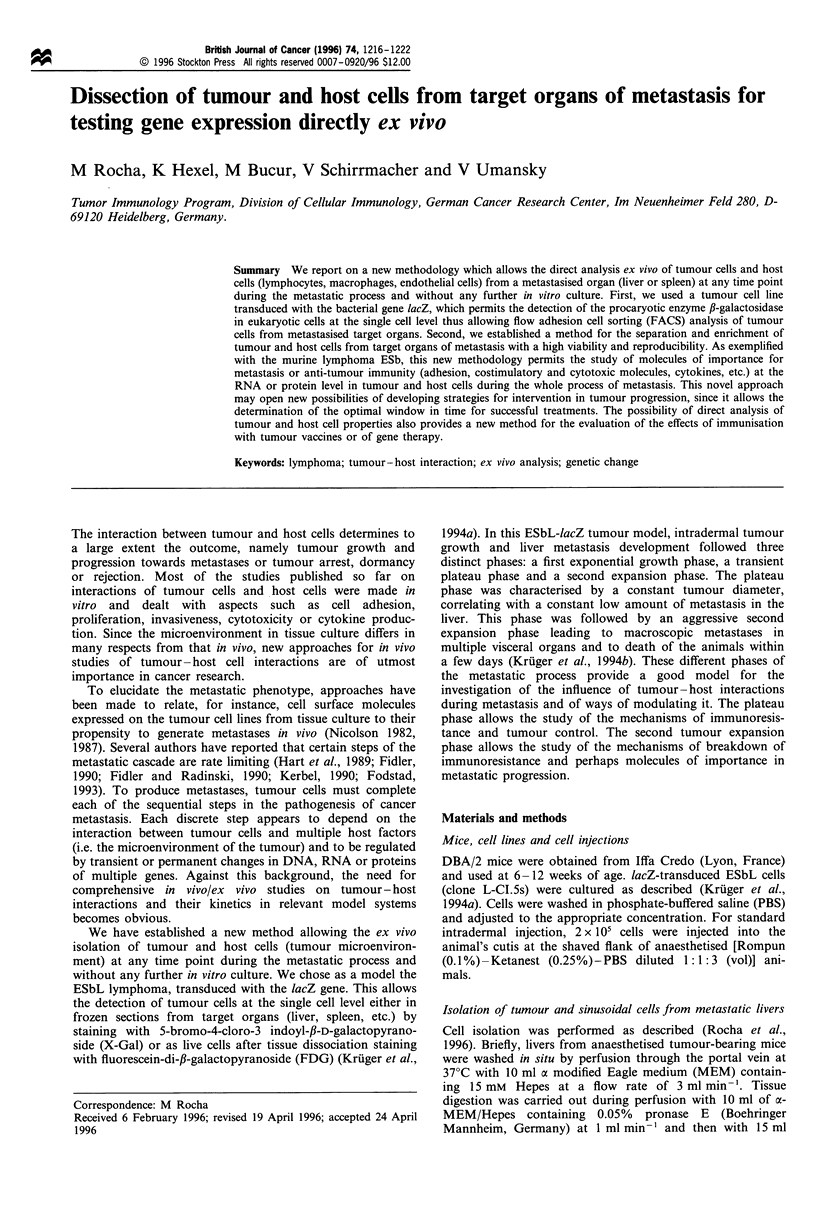

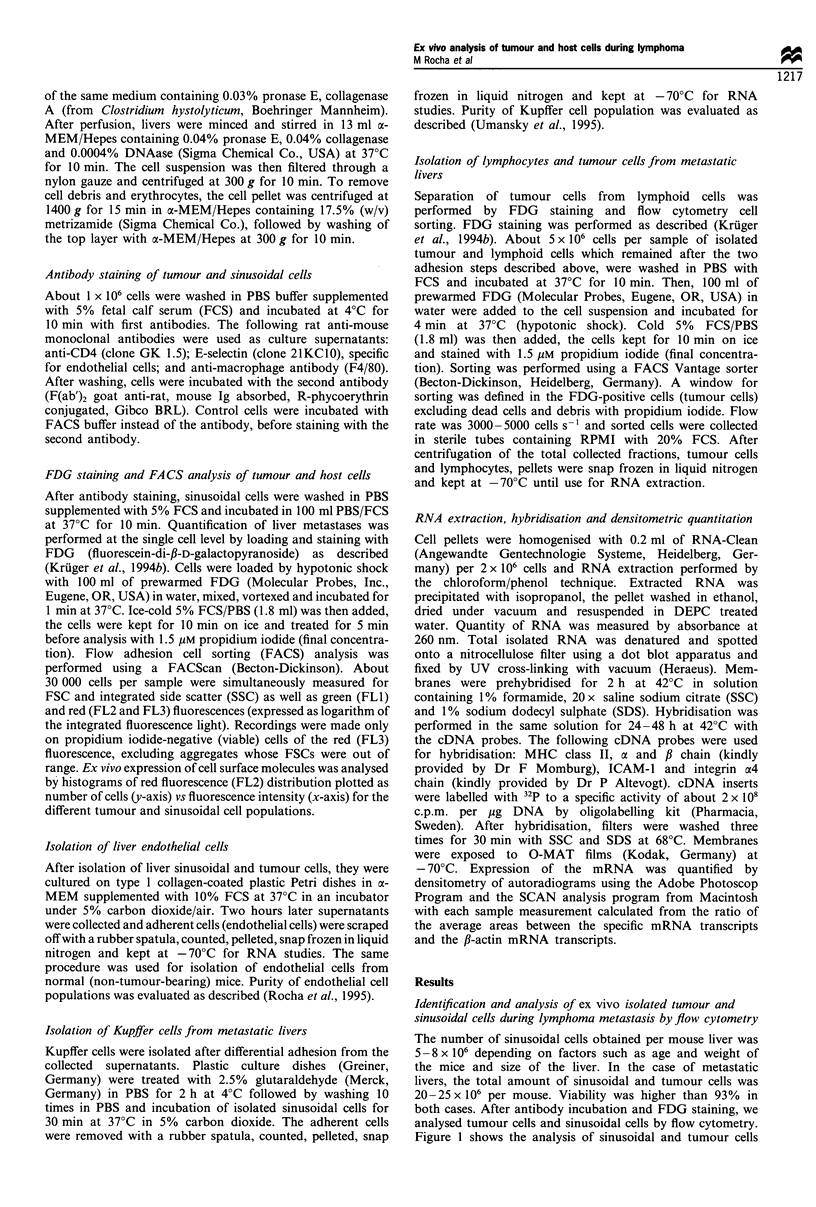

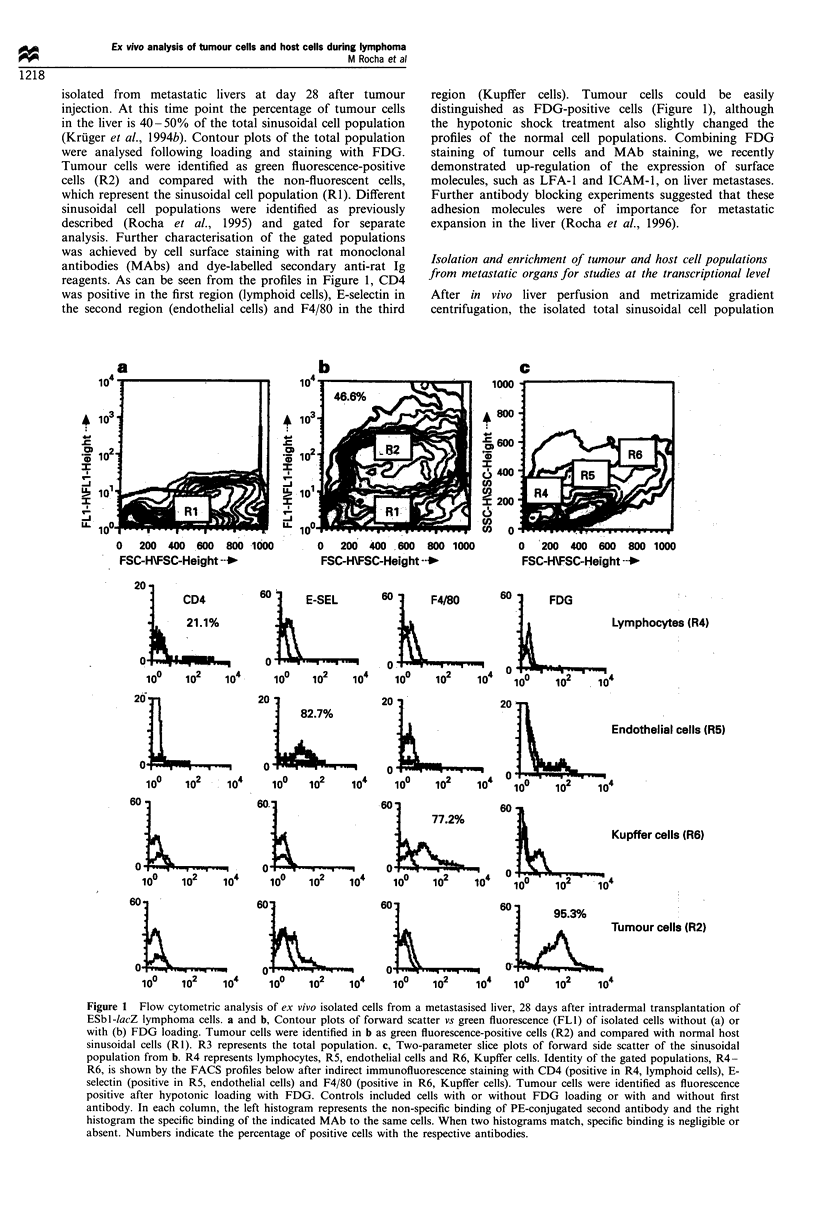

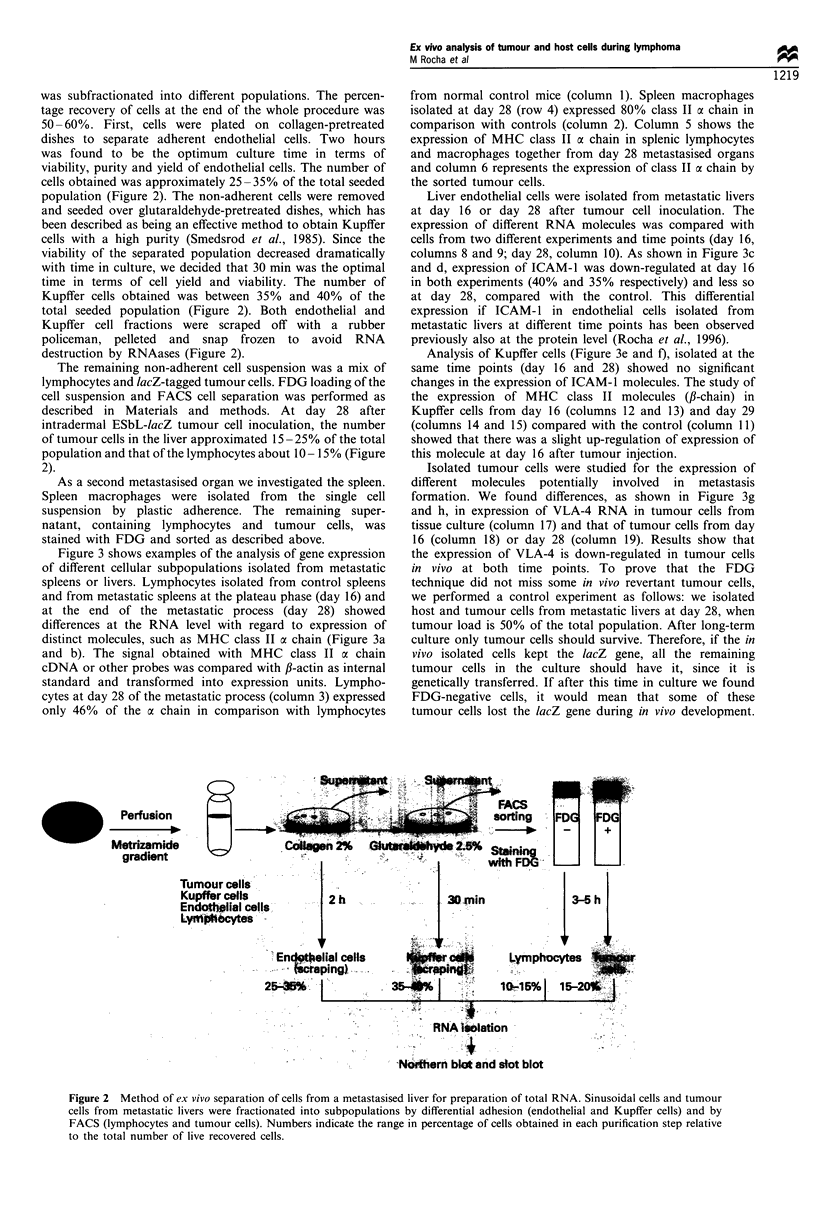

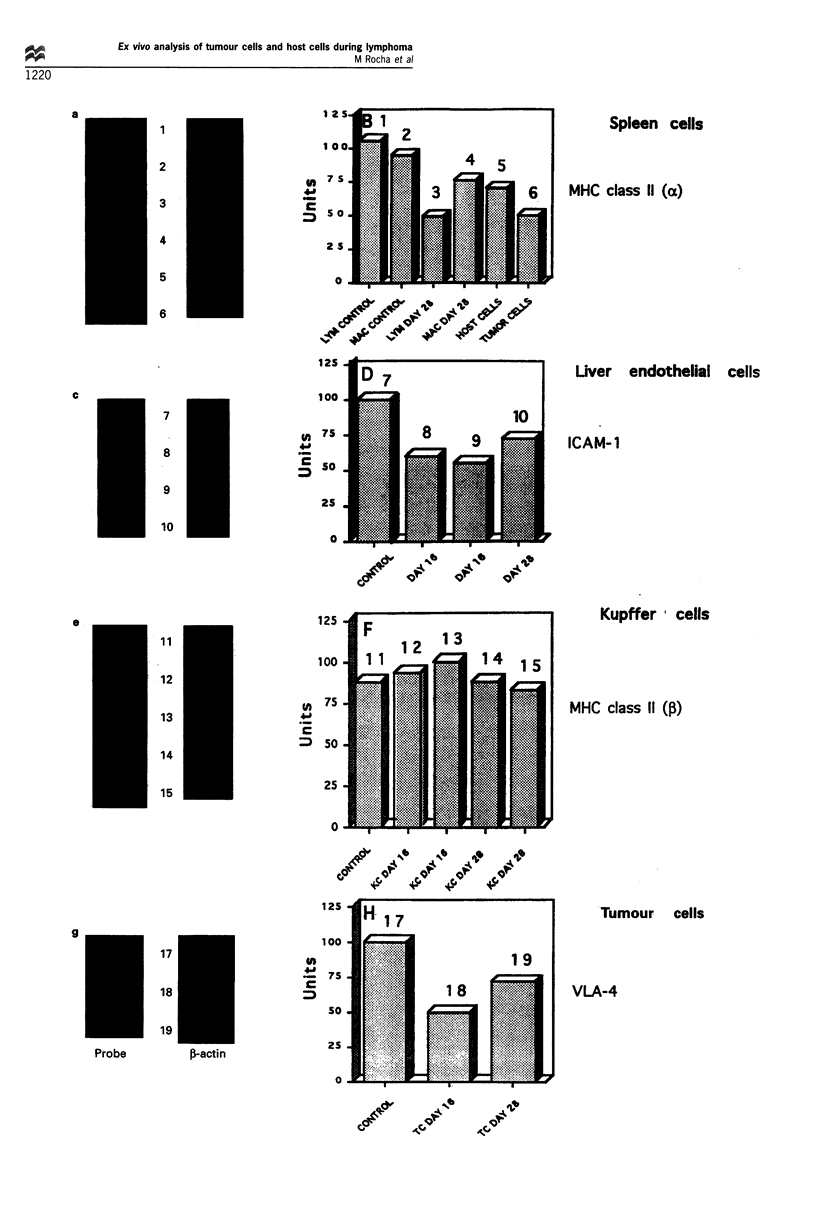

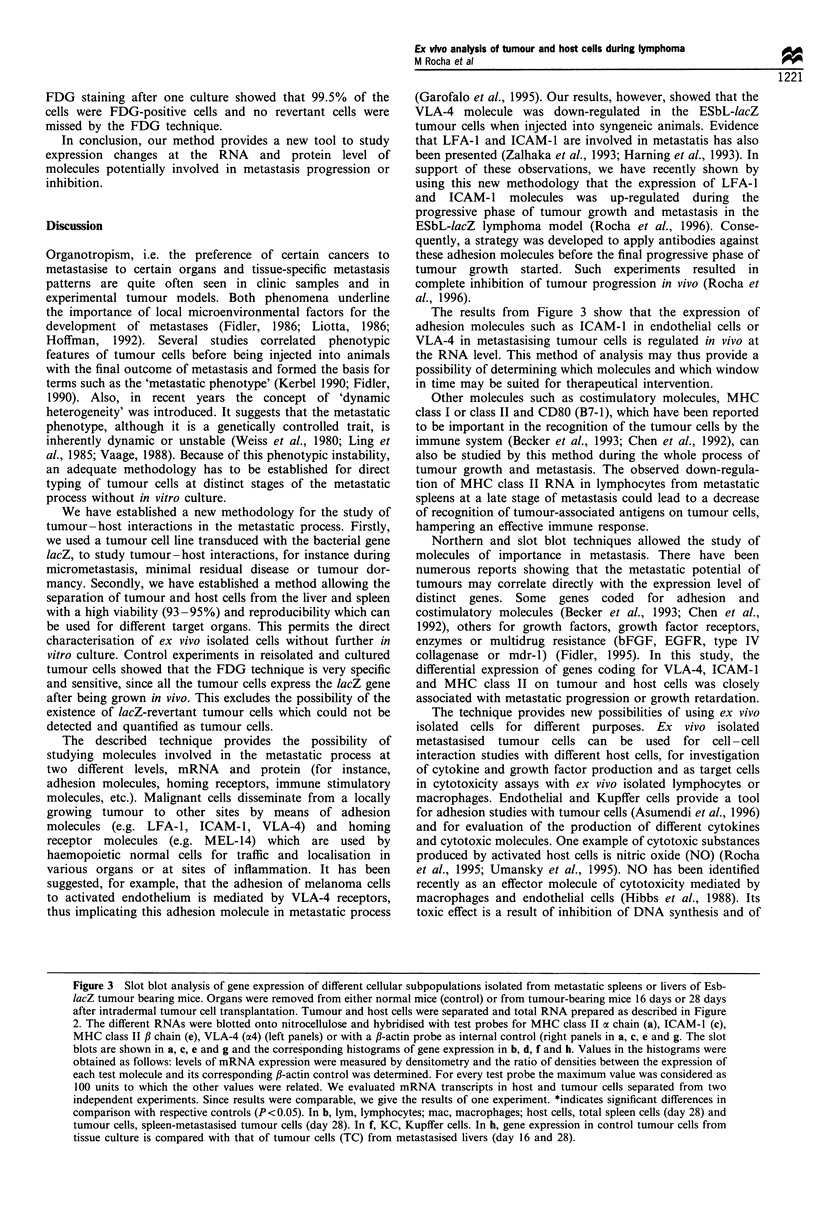

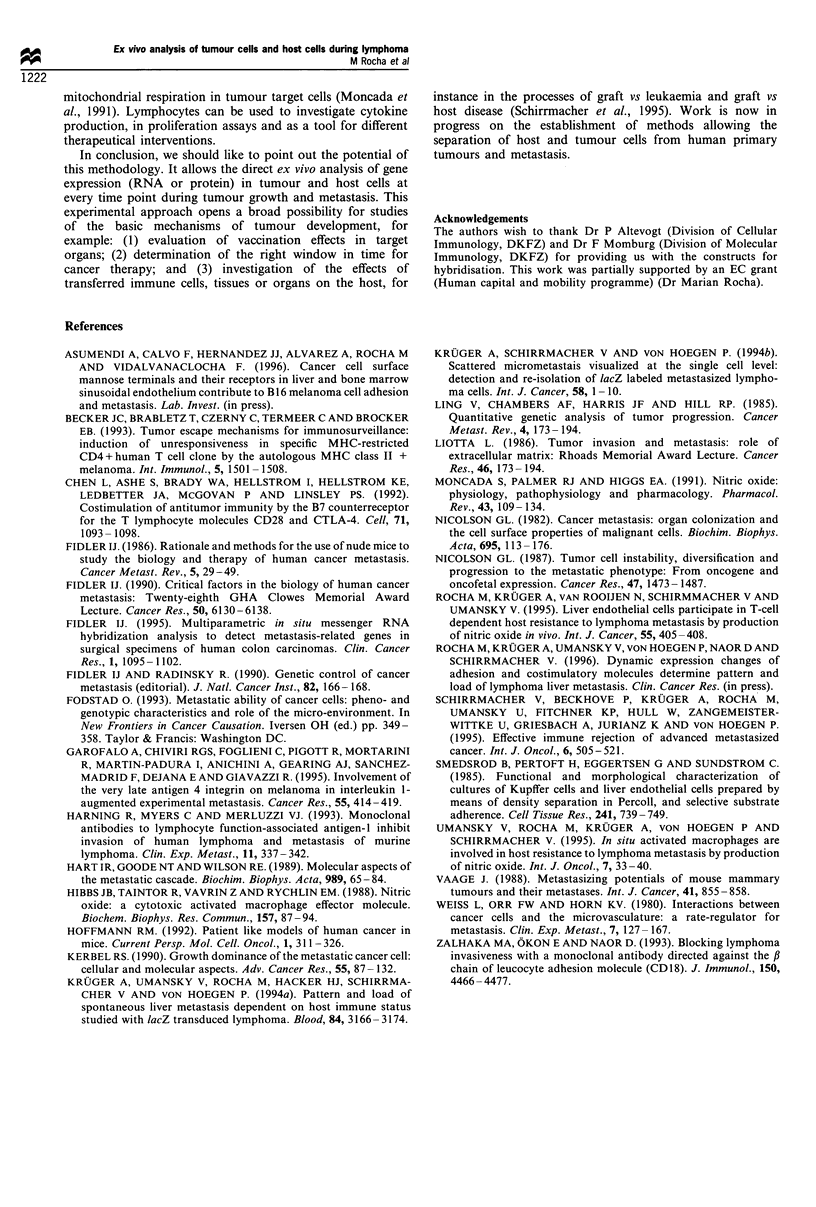


## References

[OCR_00859] Becker J. C., Brabletz T., Czerny C., Termeer C., Bröcker E. B. (1993). Tumor escape mechanisms from immunosurveillance: induction of unresponsiveness in a specific MHC-restricted CD4+ human T cell clone by the autologous MHC class II+ melanoma.. Int Immunol.

[OCR_00869] Chen L., Ashe S., Brady W. A., Hellström I., Hellström K. E., Ledbetter J. A., McGowan P., Linsley P. S. (1992). Costimulation of antitumor immunity by the B7 counterreceptor for the T lymphocyte molecules CD28 and CTLA-4.. Cell.

[OCR_00880] Fidler I. J. (1990). Critical factors in the biology of human cancer metastasis: twenty-eighth G.H.A. Clowes memorial award lecture.. Cancer Res.

[OCR_00891] Fidler I. J., Radinsky R. (1990). Genetic control of cancer metastasis.. J Natl Cancer Inst.

[OCR_00873] Fidler I. J. (1986). Rationale and methods for the use of nude mice to study the biology and therapy of human cancer metastasis.. Cancer Metastasis Rev.

[OCR_00902] Garofalo A., Chirivi R. G., Foglieni C., Pigott R., Mortarini R., Martin-Padura I., Anichini A., Gearing A. J., Sanchez-Madrid F., Dejana E. (1995). Involvement of the very late antigen 4 integrin on melanoma in interleukin 1-augmented experimental metastases.. Cancer Res.

[OCR_00908] Harning R., Myers C., Merluzzi V. J. (1993). Monoclonal antibodies to lymphocyte function-associated antigen-1 inhibit invasion of human lymphoma and metastasis of murine lymphoma.. Clin Exp Metastasis.

[OCR_00914] Hart I. R., Goode N. T., Wilson R. E. (1989). Molecular aspects of the metastatic cascade.. Biochim Biophys Acta.

[OCR_00918] Hibbs J. B., Taintor R. R., Vavrin Z., Rachlin E. M. (1988). Nitric oxide: a cytotoxic activated macrophage effector molecule.. Biochem Biophys Res Commun.

[OCR_00925] Kerbel R. S. (1990). Growth dominance of the metastatic cancer cell: cellular and molecular aspects.. Adv Cancer Res.

[OCR_00883] Kitadai Y., Ellis L. M., Takahashi Y., Bucana C. D., Anzai H., Tahara E., Fidler I. J. (1995). Multiparametric in situ messenger RNA hybridization analysis to detect metastasis-related genes in surgical specimens of human colon carcinomas.. Clin Cancer Res.

[OCR_00932] Krüger A., Umansky V., Rocha M., Hacker H. J., Schirrmacher V., von Hoegen P. (1994). Pattern and load of spontaneous liver metastasis dependent on host immune status studied with a lacZ transduced lymphoma.. Blood.

[OCR_00943] Ling V., Chambers A. F., Harris J. F., Hill R. P. (1985). Quantitative genetic analysis of tumor progression.. Cancer Metastasis Rev.

[OCR_00953] Moncada S., Palmer R. M., Higgs E. A. (1991). Nitric oxide: physiology, pathophysiology, and pharmacology.. Pharmacol Rev.

[OCR_00956] Nicolson G. L. (1982). Cancer metastasis. Organ colonization and the cell-surface properties of malignant cells.. Biochim Biophys Acta.

[OCR_00963] Nicolson G. L. (1987). Tumor cell instability, diversification, and progression to the metastatic phenotype: from oncogene to oncofetal expression.. Cancer Res.

[OCR_00969] Rocha M., Krüger A., Van Rooijen N., Schirrmacher V., Umansky V. (1995). Liver endothelial cells participate in T-cell-dependent host resistance to lymphoma metastasis by production of nitric oxide in vivo.. Int J Cancer.

[OCR_00998] Vaage J. (1988). Metastasizing potentials of mouse mammary tumors and their metastases.. Int J Cancer.

[OCR_01002] Weiss L., Orr F. W., Honn K. V. (1989). Interactions between cancer cells and the microvasculature: a rate-regulator for metastasis.. Clin Exp Metastasis.

[OCR_01007] Zahalka M. A., Okon E., Naor D. (1993). Blocking lymphoma invasiveness with a monoclonal antibody directed against the beta-chain of the leukocyte adhesion molecule (CD18).. J Immunol.

